# HGF and MET: From Brain Development to Neurological Disorders

**DOI:** 10.3389/fcell.2021.683609

**Published:** 2021-06-09

**Authors:** Claudia Desole, Simona Gallo, Annapia Vitacolonna, Francesca Montarolo, Antonio Bertolotto, Denis Vivien, Paolo Comoglio, Tiziana Crepaldi

**Affiliations:** ^1^Department of Oncology, University of Turin, Turin, Italy; ^2^Candiolo Cancer Institute, FPO-IRCCS, Candiolo, Italy; ^3^Neuroscience Institute Cavalieri Ottolenghi, Orbassano, Italy; ^4^Neurobiology Unit, Neurology, CReSM (Regional Referring Center of Multiple Sclerosis), San Luigi Gonzaga University Hospital, Orbassano, Italy; ^5^Department of Molecular Biotechnology and Health Sciences, University of Turin, Turin, Italy; ^6^INSERM U1237, University of Caen, Gyp Cyceron, Caen, France; ^7^Department of Clinical Research, Caen-Normandie University Hospital, Caen, France; ^8^IFOM, FIRC Institute for Molecular Oncology, Milan, Italy

**Keywords:** HGF, MET, synaptogenesis, autism, cerebral ischemia, spinal cord injury, amyotrophic lateral sclerosis, multiple sclerosis

## Abstract

Hepatocyte growth factor (HGF) and its tyrosine kinase receptor, encoded by the MET cellular proto-oncogene, are expressed in the nervous system from pre-natal development to adult life, where they are involved in neuronal growth and survival. In this review, we highlight, beyond the neurotrophic action, novel roles of HGF-MET in synaptogenesis during post-natal brain development and the connection between deregulation of MET expression and developmental disorders such as autism spectrum disorder (ASD). On the pharmacology side, HGF-induced MET activation exerts beneficial neuroprotective effects also in adulthood, specifically in neurodegenerative disease, and in preclinical models of cerebral ischemia, spinal cord injuries, and neurological pathologies, such as Alzheimer’s disease (AD), amyotrophic lateral sclerosis (ALS), and multiple sclerosis (MS). HGF is a key factor preventing neuronal death and promoting survival through pro-angiogenic, anti-inflammatory, and immune-modulatory mechanisms. Recent evidence suggests that HGF acts on neural stem cells to enhance neuroregeneration. The possible therapeutic application of HGF and HGF mimetics for the treatment of neurological disorders is discussed.

## Introduction

Hepatocyte growth factor (HGF) is a pleiotropic cytokine isolated as a potent cell motility factor for epithelial cells (Stoker et al., 1987) and as a strong mitogen for hepatocytes in primary cultures (Nakamura et al., 1989). HGF, upon binding to its receptor, encoded by the proto-oncogene MET (Naldini et al., 1991), elicits relevant biological activities, including cell motility, division, survival, and differentiation (Trusolino et al., 2010). MET is primarily expressed not only in epithelial and endothelial cells (Gentile et al., 2008) but also in myoblasts and neuronal precursors, contributing to the development of muscle and nervous structures (Bladt et al., 1995; Uehara et al., 1995; Maina et al., 1996, 1997). The expression and functions of *HGF* and *MET* are fundamental during embryogenesis. Indeed, in Hgf- or Met-null mice, the development of liver is severely impaired and the placental labyrinth is hypomorphic, leading to death *in utero* (Bladt et al., 1995; Schmidt et al., 1995; Uehara et al., 1995). Moreover, ablation of these genes leads to the complete absence of hypaxial muscles (diaphragm, limbs, tongue), reflecting that HGF-MET have a pivotal role in proliferation and motility of these muscle progenitors (Birchmeier and Gherardi, 1998). Furthermore, HGF or MET ablation affects the ‘wiring’ of the nervous system, leads to a decreased survival of sensory and sympathetic neurons, and reduces axon bundling of certain motor nerves (Maina et al., 1997, 1998; Helmbacher et al., 2003).

MET activation is involved in angiogenesis, wound healing, cell scattering, proliferation, and cancer invasion (Birchmeier et al., 2003; Trusolino et al., 2010). Exhaustive information on the functions of HGF-MET outside the nervous system can be found in recent reviews (Gallo et al., 2014b, 2015; Comoglio et al., 2018).

In this review, we focus on the genetic, molecular, and cellular mechanisms driven by HGF-MET in the nervous system, starting from their involvement in brain development and ending to their implication in neurodevelopmental disorders (NDDs). We then discuss the favorable effect of HGF in promoting neuron survival in the context of brain injury and in neurodegenerative disorders, pointing out its pro-angiogenic, anti-fibrotic, anti-inflammatory, immune-modulatory, and neuro-regenerative properties. We finally emphasize the promising therapeutic potential of HGF and HGF mimetics enlightened by a panel of animal models and by the first human clinical trials.

## Domain Structure and Signaling Components of HGF-MET Pair

**FIGURE 1:**
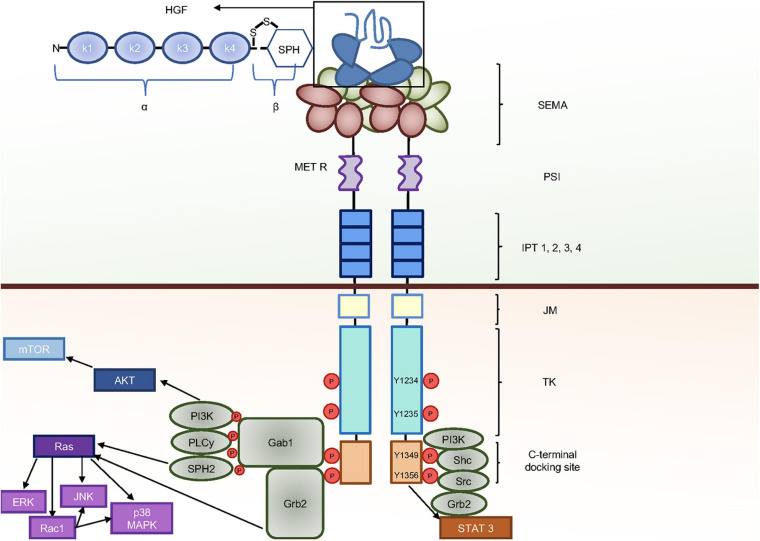
HGF-MET and molecular signaling components. Upon HGF binding, MET forms dimers and transphosphorylates on the two catalytic tyrosines Y1234 and Y1235 and the two carboxyterminal tyrosines Y1349 and Y1356, which provide a docking platform for the binding of adaptor and effector signaling proteins. Different downstream pathways are then activated, leading to a variety of biological activities.

Hepatocyte growth factor is produced and secreted as pro-HGF, representing the inactive precursor, and then activated by proteolytic processing in the extracellular milieu (Naldini et al., 1992). The active cleaved HGF is a heterodimer made by two disulfide bridge-linked chains, namely, the heavy and light chains (α and β, respectively) (Figure 1). The HGF protein contains six plasminogen-related structural domains: one short N-terminal domain and four ‘kringle’ domains (K1–K4) in the α chain. A serine proteinase homology (SPH) domain encompasses the β chain. However, the SPH domain lacks proteolytic activity due to a mutation of the catalytic residues. HGF is the only ligand for the tyrosine kinase receptor encoded by the MET proto-oncogene (Bottaro et al., 1991; Naldini et al., 1991). MET is synthesized as a single-chain precursor, which is then cleaved into an α, β heterodimer by a protease of the furin family in the Golgi apparatus (Komada et al., 1993). The MET α and the N-terminus of the β chain are disulfide linked and assemble to generate the HGF binding domain. Structurally, the N-terminus is also composed of one PSI and four IPT domains (Gherardi et al., 2003, 2006; Basilico et al., 2008). The β chain continues with a trans-membrane and an intracellular domain. The latter is composed of a juxta-membrane, a tyrosine kinase, and a C-terminal domain (Figure 1). Upon HGF binding to MET, the receptor is activated by dimerization. The activation occurs via *trans*-phosphorylation of two catalytic tyrosines (Y1234 and Y1235) located in the kinase domain, followed by the subsequent auto-phosphorylation of the two docking tyrosines (Y1349 and Y1356) in the C-terminal tail. The multifunctional docking site of MET recruits to the plasma membrane stereotypical adaptor/signaling molecules such as Gab1, Grb2, SPH2, phosphoinositide-3-kinase (PI-3K), Src, phospholipase c gamma (PLCγ), Shc, and signal transducer and activator of transcription 3 (STAT3) (Ponzetto et al., 1994; Weidner et al., 1996; Schaeper et al., 2000; Comoglio et al., 2018; Figure 1). These events trigger downstream signaling pathways, including AKT, Ras (rat sarcoma), and STAT3 pathways, which are required for MET biological activities (Ponzetto et al., 1994; Trusolino and Comoglio, 2002; Birchmeier et al., 2003; Trusolino et al., 2010). AKT activates mammalian target of rapamycin (mTOR), promoting protein synthesis and cell growth. Ras leads to the activation of different mitogen-activated protein kinase (MAPK) cascades, including extracellular-signal-regulated kinase (ERK), p38^*MAPK*^, and c-Jun N-terminal protein kinase (JNK) (Graziani et al., 1993). Moreover, Ras induces Ras-related C3 botulinum toxin substrate 1 (Rac1), which in turn leads to further stimulation of p38^*MAPK*^ and JNK (Trusolino et al., 2010). ERK is mainly implicated in cell proliferation and Rac1 in cell migration, while p38^*MAPK*^ and JNK are involved in differentiation and apoptosis. STAT3 activation is essential for tubulogenesis (Boccaccio et al., 1998). Nevertheless, the contribution of distinct signals to specific biological events is dependent on context in which these signals operate, varying according to the cell types.

## HGF Is a Neurotrophic Factor and an Axonal Chemoattractant in the Embryonic Development

A large body of evidence suggests that HGF is a neurotrophic factor for neurons. Initial studies were performed on mouse embryos with targeted disruption of either *Hgf* or *Met* locus (Ebens et al., 1996; Maina et al., 1997, 1998). However, these studies are limited to early stages of embryo development, as mice lacking the HGF or MET receptor die during embryogenesis (E14–E15). Mouse embryos possessing a non-functional MET receptor result in short, less branched spinal sensory nerves and apoptotic sympathetic neuroblasts *in vivo* (Maina et al., 1997, 1998; Maina and Klein, 1999). Moreover, HGF cooperates with nerve growth factor (NGF) *in vitro* to enhance the axonal growth of dorsal root ganglion sensory neurons (Maina et al., 1997) and sympathetic neurons (Maina et al., 1998). Notably, HGF is implicated in guiding spinal motoneurons to their target muscles in the limbs (Ebens et al., 1996). Several studies indicate that HGF promotes survival and axon outgrowth of specific sub-populations of motoneurons (Yamamoto et al., 1997; Novak et al., 2000; Helmbacher et al., 2003; Lamballe et al., 2011; Caruso et al., 2014) and guides cranial and vagus motor axons to the pharyngeal arches (Caton et al., 2000; Isabella et al., 2020).

HGF-MET transcripts and proteins were spatially and temporally mapped on rodent brains using various approaches including immunohistochemistry, Western blotting, and *in situ* hybridization. Studies in mice show that HGF and MET expression is detectable in the brain during early embryonic growth at E12 and E13, respectively (Jung et al., 1994; Achim et al., 1997; Thewke and Seeds, 1999). Expression of both HGF and MET is first found in the cortical ventricular zone and later in the cortical plate (Powell et al., 2001). HGF is also expressed in the proliferative zone of the ganglionic eminence (Powell et al., 2001). Mice with inactivation of the urokinase plasminogen activator receptor (uPAR) gene, a key component of the HGF activation (Blasi, 1993), show diminished levels of HGF in cortical interneurons beginning at E16.5, indirectly suggesting that HGF has motogenic effects also on cortical interneuron precursors (Powell et al., 2001). Moreover, olfactory interneuron precursors are chemoattracted by HGF from the sub-ventricular zone (SVZ) of the lateral ventricles, through the rostral migratory stream, to the olfactory bulb (Giacobini et al., 2007; Garzotto et al., 2008). HGF and MET are simultaneously expressed during cerebellum development, which occurs prevalently post-natally. Indeed, HGF increases the proliferation of cerebellum granule cell precursors and this effect is reduced in a hypomorphic MET mutant (Grb2-binding incompetent) that leads to a smaller size of the cerebellum (Ieraci et al., 2002).

A number of studies independently described that HGF is neurotrophic for embryonic cortical neurons cultured *in vitro*. HGF increases the numbers of hippocampal neurons and the length of their dendrites (Honda et al., 1995; Lim and Walikonis, 2008). Specific neurotrophic effects of HGF were reported on calbindin-D containing hippocampal neurons (Korhonen et al., 2000) and dopaminergic mesencephalic neurons (Hamanoue et al., 1996; Lan et al., 2008).

## Temporal and Spatial Expression of HGF-MET in the Postnatal Developing Brain

**FIGURE 2:**
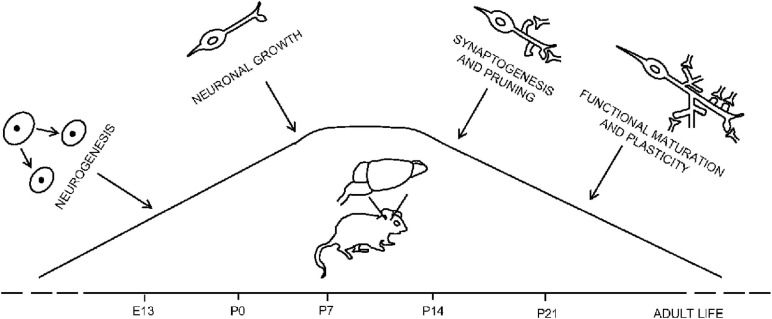
Temporal and spatial regulation of MET levels in the mouse brain. MET expression increases during the perinatal period and peaks in correspondence of the processes of neurite growth and synaptogenesis (P7–P14) to decrease when brain circuits go through maturation and synaptic plasticity (P21).

Sites of HGF and MET expression in the postnatal brain include the CA-1 area of hippocampus and limited regions of the cortex (Jung et al., 1994; Thewke and Seeds, 1999; Judson et al., 2011). MET transcripts levels in the cerebral cortex are quite low at pre-natal stage and increase significantly starting from post-natal day P0 to peak at P7 (Figure 2). Since then, MET levels remain elevated in the neocortex, hippocampus, and sub-cortical limbic regions of mice forebrains during the second post-natal week, decreasing from P21 to adult age (Judson et al., 2009). Interestingly, there is a coincidence between MET peak expression and the early and late stages of neurodevelopmental processes such as dendritic morphology, neurite outgrowth, and synaptogenesis (Gutierrez et al., 2004; Judson et al., 2009, 2011; Qiu et al., 2014). Exogenous HGF enhances cortical pyramidal dendrite arborization in organotypic slice cultures of mouse somatosensory cortex at P6 and P7 (Gutierrez et al., 2004). MET expression can be detected at a sub-cellular level in the growth-cone of P0 mice developing cortical tissue as well as in cultured hippocampal neurons, suggesting a functional role mediated by MET in neuronal growth (Peng et al., 2016). In line with this work, MET is enriched, during forebrain formation, in developing axon tracts and in the neuropil, mostly in pre- and post-synaptic compartments, mirroring an active transport to the synapse (Judson et al., 2009; Eagleson et al., 2013). The HGF transcript is also detected in the neuropil. Despite that, phospho-MET (pMET) is not detected in axon tracts, while it is enriched in the neuropil (Eagleson et al., 2016). Moreover, MET expression levels in mice brain preparations of the *striatum radiatum* layer of hippocampal CA1 region are comparable in pre- and post-synaptic terminals at P7 peak in mouse. At a later stage, MET is predominantly pre-synaptic, reflecting a dynamic regulation of MET expression depending on the post-natal age of the examined mice (Eagleson et al., 2013).

The pMET protein levels peak at P7–P14 in the neocortex, a period corresponding to synaptogenesis and dendritic outgrowth, and are downregulated during the 3rd post-natal week, between P14 and P17, when pruning and functional synapse maturation occur (Eagleson et al., 2016). A comparative analysis on primates (*Rhesus* monkey) (Judson et al., 2011) reveals a similar temporal expression pattern of MET as in rodents, with peak levels corresponding to the same periods detected in mice and rats. Altogether, these studies raised new putative roles for the MET receptor in synaptogenesis.

## Beyond Neurotrophic Functions: HGF-MET Play a Role in Synaptogenesis and Neuroplasticity

Circumstantial evidence indicates that MET is implicated in the synapse formation. MET forms clusters at excitatory synapses in relatively mature cultured hippocampal neurons and co-localizes with the post-synaptic protein PSD-95 and with the glutamatergic N-methyl-D-aspartate (NMDA) receptor subunit GluN2B (Tyndall and Walikonis, 2006). This observation may appear to contradict the finding that MET levels are reduced in mature circuits (Judson et al., 2009; Peng et al., 2016). However, a more localized synaptic expression of MET in the adult brain was indeed shown (Eagleson et al., 2013). MET is enriched in the post-synaptic density fraction, and HGF administration induces MET phosphorylation and clustering of synaptic proteins such as GluN2B, CAMK-II, and the glutamatergic α-amino-3-hydroxy-5-methyl-4-isoxazolepropionic acid (AMPA) receptor GluA1, suggesting a functional connection between glutamatergic synapse and MET signaling (Tyndall and Walikonis, 2006, 2007). MET activation is thus involved in brain plasticity, as suggested by the fact that HGF administration on hippocampal slices enhances the phosphorylation of the glutamatergic NMDA receptor subunit GluN1, augments the NMDA-mediated currents, and increases the amplitude of long-term potentiation (LTP) induced by elevated neuronal activity (Akimoto et al., 2004). Accordingly, HGF-MET axis contributes to the activity-dependent regulation of physiological learning and memory performance in the adult brain (Kato et al., 2012). Qiu et al. recently employed complementary *in vitro* and *in vivo* methods to examine how altered MET signaling impacts synaptic development, observing an opposite effect on neuronal morphology in a stage-dependent way. Accordingly, MET signaling enhancement in primary hippocampal neurons during the second culture week leads to dendritic growth. At later developmental stage, MET signaling leads to dendritic spine development (Qiu et al., 2014). These observations, together with the fact that spine head size and geometry are associated with AMPA receptor content and maturation status (Matsuzaki et al., 2001), suggest that MET signaling may control many aspects of glutamatergic synapse development, including the timing of excitatory synapse maturation (Qiu et al., 2014). Furthermore, conditional knockout (cKO) of MET in forebrain excitatory neurons reduces dendritic complexity in mice and correlates with an earlier functional maturation of the hippocampal CA3 > CA1 synapse and a disrupted intracortical connectivity phenotype in frontal cortex (Qiu et al., 2011). At a molecular level, MET cKO mice showed altered glutamate receptor expression profiles in the hippocampus at P14, including increased GluA1, GluN2A, and decreased GluN2B at the synaptic sites.

MET ablation during hippocampus development alters the time course of the LTP and long-time depression (LTD) development, the two major forms of synaptic plasticity. An enhancement of LTP and LTD at early developmental stage (P12–P14) at the hippocampal CA1 axons is observed compared to wild-type mice. The opposite effect occurs at the adult stage (P56–P70), during which wild-type mice show robust LTP and LTD compared to MET cKO mice in which LTP and LTD are markedly decreased. Thus, MET signaling is implicated not only in neuronal growth and maturation but also in the timing regulation of synaptic plasticity during forebrain glutamatergic circuit development (Ma et al., 2019).

Mechanistically, HGF-induced synapse formation is mediated by PI-3K and not MAPK activation, as inhibition of the PI-3K/AKT pathway affects synaptogenesis, while blocking MAPK/ERK does not decrease synapse density and only affects dendritic growth (Eagleson et al., 2016). A recent study by Peng et al. describes, for the first time, that MET activation leads to stimulation of Rho family small GTPase cdc42 in a PI-3K-dependent way (Peng et al., 2016). Rho small GTPase family (rac1, cdc42, and rhoA) has an established role in mediating neurite growth and spine morphogenesis (Tashiro et al., 2000; Kasri et al., 2009; Ba et al., 2013). Accordingly, MET-mediated cdc42 activation is associated with enhanced dendritic growth and spine morphogenesis.

## Implications of HGF-MET Gene Mutations in NDDs

MET has an important role in neuron architecture construction and synapse maturation, as described in previous sections. The reduced gene expression or deletion of MET *in vivo* in mice results in altered synapse maturation (reviewed in Ma and Qiu, 2019). A strong evidence of HGF-MET axis importance in the human development of nervous system comes from some studies that reveal a genetic implication of HGF and MET genes in some NDDs such as autism spectrum disorder (ASD), schizophrenia, and non-syndromic hearing loss. These disorders are all associated with synaptic and neuronal circuit disruption.

**FIGURE 3:**
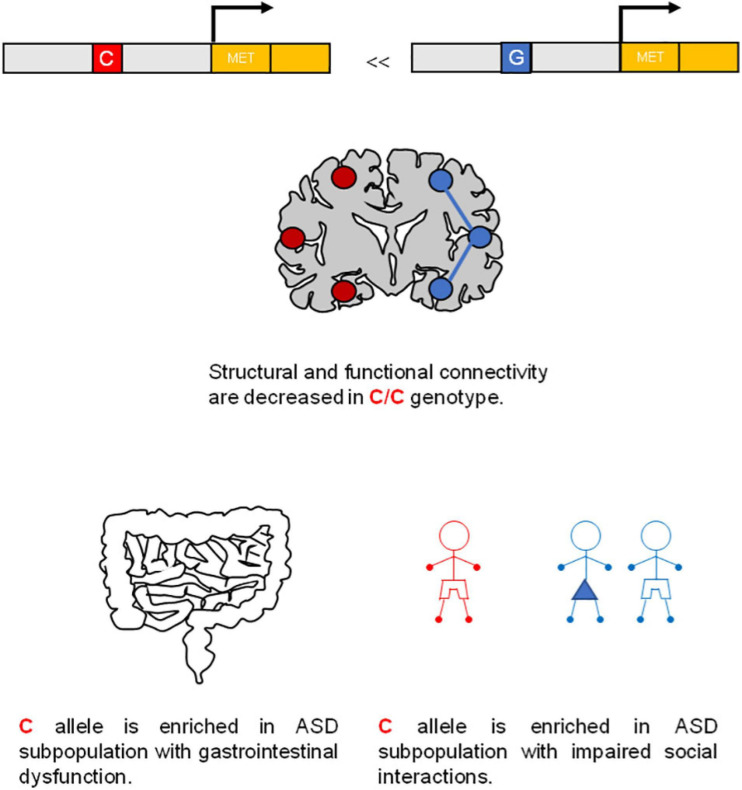
*MET* gene is a risk factor for autism spectrum disorder (ASD). A common C allele (rs1858830 “C”) on MET promoter region is a risk factor for ASD.

Autism spectrum disorder is associated with deficits in the ontogeny of neural circuits (Geschwind and Levitt, 2007). The gene coding for human MET (Online Mendelian Inheritance in Man Code: 164860) is localized within the chromosome 7q31, which represents a common ASD linkage region. Campbell et al. (2006) reported a genetic association of a common “C” allele (rs1858830 “C”) in the *MET* promoter region in more than 200 families with ASD (Figure 3). This single-nucleotide polymorphism (SNP) variant leads to a 50% decrease in MET transcription, linked to ASD risk. In particular, this variant produces an altered fixation of SP1 and PC4 transcription factors in the promoter, resulting in a decreased transcription activity (Campbell et al., 2006). Two other genetic studies revealed a positive association between ASD and other SNPs of MET in the same intron (intron 1), such as rs38845 in Caucasian population patients (Sousa et al., 2009) and rs38841 in Japanese population patients (Thanseem et al., 2010). Thus, independently on the genetic variation, these studies reveal a genetic association between MET and ASD. In addition, the transcription of the human *MET* gene can also be regulated by FOXP2 and MeCP2, which are both factors that affect ASD-related circuit development in humans (Mukamel et al., 2011; Plummer et al., 2013). The connection between MET and ASD is supported by the fact that MET is expressed in brain areas involved in higher levels of cognition, executive functions, and language skills (Judson et al., 2011; Mukamel et al., 2011).

Furthermore, MET levels in post-mortem temporal lobe samples of autistic or healthy individuals were analyzed in other studies, showing a reduction in MET transcripts and proteins in patients with ASD compared to control ones (Campbell et al., 2007). Low levels of HGF are also found in plasma of ASD patients (Sugihara et al., 2007) and children with co-occurrence of gastro-intestinal diseases. Interestingly, MET promoter variant rs1858830 signaling appears to contribute to gastro-intestinal abnormalities associated with ASD (Campbell et al., 2009). Rs1858830 MET promoter variant is also predictive of atypical functional magnetic resonance imaging (fMRI) activation and deactivation pattern of human brain to social stimuli, and it impacts functional and structural connectivity in the temporo-parietal lobes, which selectively express MET (Rudie et al., 2012; Figure 3). In line with these studies, forebrain-specific MET cKO mice show altered circuit formation and function including synaptic plasticity. Consistently, animal studies and clinical imaging have provided evidence that disrupted MET signaling produces functional as well as morphological alterations in neurons in those brain regions implicated in ASD endophenotypes (reviewed in Peng et al., 2013; Eagleson et al., 2017; Ma and Qiu, 2019).

Mutations in both HGF and MET genes are also linked to non-syndromic hearing impairment (NSHI), which is a partial or total loss of hearing that is not associated with other signs and symptoms. Three non-coding mutations in the HGF gene were found at the autosomal-recessive NSHI locus (DFNB39) in some Pakistani and Indian families (Schultz et al., 2009). Two mutations occurred in the 3’ untranslated region of an alternative splice form of HGF, while the third mutation altered splicing by affecting the relative strengths of the spliced forms of HGF gene. Furthermore, a cKO model of HGF deregulation, in which HGF was deleted from a limited number of tissues including cochlea, was associated with extensive cochlear pathology with morphogenetic defects and non-progressive deafness (Schultz et al., 2009). A rare MET missense genetic mutation (c.2575T > G p.Phe859Val) was reported in Pakistani family patients segregating with DFNB97 deafness (Mujtaba et al., 2015). Recently, another MET mutation was found in a Moroccan bilateral DFNB97 deaf patient, consisting of a homozygous missense mutation (c.948A > G; p.Ile316Met) of MET, affecting a highly conserved residue in the SEMA domain (Bousfiha et al., 2019).

A study on Caucasian population patients with schizophrenia showed a genetic association between the pathology and MET SNP variants (Burdick et al., 2010). Few studies also revealed a genetic implication of *HGF* gene in schizophrenia. The upregulation of *HGF* in schizophrenia was reported in the CommonMind Consortium (CMC) study (Fromer et al., 2016). Recently, *HGF* was found to be upregulated in a study in which the authors used fibroblasts collected from a unique population of schizophrenia patients in northern Sweden (Etemadikah et al., 2020).

## HGF Protects Neurons From Death

**FIGURE 4:**
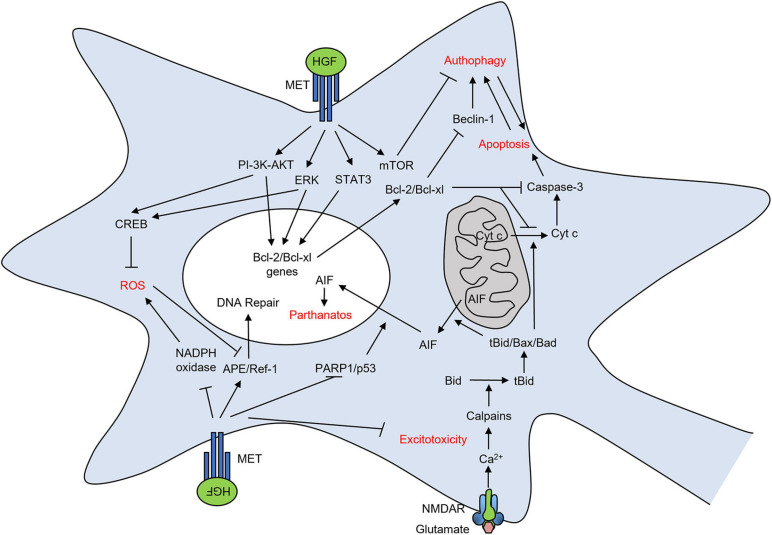
HGF-MET pair protects neurons from death after brain damage. Representation of signaling mechanisms through which HGF-MET prevents oxidative stress, excitotoxicity, apoptosis, autophagy, and parthanatos in neurons.

Hepatocyte growth factor and its MET receptor exert important trophic effects in neurons of the central and peripheral nervous system during development (see above). These protective functions are likely rewired in many neurological and neurodegenerative disorders in the adult. Different insults induce brain tissue damage and lead to neuronal cell death such as ischemic injury, oxidative stress, and glutamate toxicity (Figure 4). Indeed, a large body of evidence suggests a key role of HGF-MET in the multifaceted protective mechanisms against neuron damage.

The expression of both HGF and MET receptor mRNA is strongly increased in response to cerebral ischemic injury (Honda et al., 1995). HGF improves the neurological consequences of stroke by reducing the infarct volume of the ischemic brain and by preventing neuronal death through the reduction of apoptosis (Miyazawa et al., 1998; Tsuzuki et al., 2001; Shimamura et al., 2004).

Hepatocyte growth factor anti-apoptotic effects are mediated by the PI-3K/AKT pathway in rat cerebellar granule cells *in vitro* in an ERK-independent manner (Zhang et al., 2000). In cortical neurons subjected to hypoxia/reoxygenation, HGF protects from apoptosis via the ERK pathway and, at a lesser extent, the PI-3K/AKT pathway (He et al., 2008). In the context of brain ischemic injury *in vivo*, HGF inhibits apoptosis via transient activation of ERK (Niimura et al., 2006a). The induction of neuroprotective genes, such as Bcl-2 and Bcl-xl, also contributes to HGF inhibition of apoptosis (Tsuzuki et al., 2000, 2001; He et al., 2008) and is promoted by STAT3 activation (Nakagami et al., 2001; Qiu et al., 2005; Dziennis and Alkayed, 2008). The implication of the MET/STAT3/Bcl-2 pathway in HGF protection from apoptotic neuronal death in stroke has been recently suggested in a rat model of transient middle cerebral artery occlusion (tMCAO) (Tang et al., 2020).

Interestingly, HGF also reduces a caspase-independent ischemic neuronal cell death by preventing apoptosis-inducing factor (AIF) translocation downstream of poly(ADP-ribose)polymerase 1 (PARP1) and p53 (Cregan et al., 2004; Niimura et al., 2006b). This work suggests that HGF-MET may also protect from parthanatos, a form of caspase-independent regulated cell death occurring especially in neurons and driven by PARP1 hyperactivation (Galluzzi et al., 2018).

Oxidative DNA stress occurring after cerebral ischemia is associated with a decrease in apurinic/apyrimidinic endonuclease/redox factor-1 (APE/Ref-1) expression (Fujimura et al., 1999). APE/Ref-1 is a multifunctional protein in the DNA base repair pathway that is responsible for repairing apurinic/apyrimidinic sites in DNA after oxidation (Fritz et al., 2003). Interestingly, HGF counteracts the oxidative DNA damage after cerebral ischemia by increasing the level of APE/Ref-1-positive cells in the hippocampal CA1 region (Niimura et al., 2006c). In the same hippocampal region, and after cerebral ischemia, HGF also decreases the activity of the NADPH oxidase and consequent ROS generation in glia-like cells (Niimura et al., 2006c). In line with this work, HGF decreases oxidative stress in an Alzheimer’s disease (AD) transgenic mouse model (Takeuchi et al., 2008). The authors suggest an implication of the phosphorylated CREB, mediated by PI-3K and MAPK signaling pathways, that is known to decrease neurotoxicity and oxidative stress-induced neuronal death (Du and Montminy, 1998; Riccio et al., 1999; Zou and Crews, 2006).

A crosstalk between apoptosis and autophagy exists, and the interaction of anti-apoptotic regulators such as Bcl-2 and Bcl-xl suppresses the autophagy promoter Beclin-1 activity, resulting in autophagy inhibition (Pattingre et al., 2005). Autophagy is a phagocytic degradation process whose deregulation may contribute to neuronal cell death and is involved in the mechanisms of pathologic conditions, such as cerebral ischemia (Galluzzi et al., 2016; Sun et al., 2019). HGF protects neurons in tMCAO rats through the inhibition of apoptosis and autophagy. Mechanistically, neuronal death protection from autophagy seems to involve the MET downstream mTOR signaling, whose activation (phosphorylation) is enhanced after tMCAO (Shang et al., 2010).

Among the neurotoxic mechanisms leading to neuronal death, a prominent role is played by the excessive stimulation of NMDA glutamatergic receptor and increased Ca^2+^ influx. Excitotoxicity is involved in many pathological conditions, such as cerebral ischemia, epilepsy, Parkinson’s disease (PD), AD, and amyotrophic lateral sclerosis (ALS) (Dingledine et al., 1999; Lewerenz and Maher, 2015). The accumulation of intracellular calcium activates the calpains that cleave Bid. The truncated Bid interacts with Bax and Bad and permeabilizes the mitochondrial membrane, freeing cytochrome c or AIF (Broughton et al., 2009). HGF reduces NMDA-induced neuronal death in cerebellar granule neurons *in vitro*. Mechanistically, the neuroprotective effect is mediated by the PI-3K activation (Hossain et al., 2002). Moreover, HGF prevents NMDA-induced neuronal death in hippocampal neuron cultures *in vitro* by blocking pro-apoptotic caspase-3 activation and translocation of AIF into the nucleus (Ishihara et al., 2005). It remains unclear to which extent the protection mediated by HGF-MET from the excitotoxic insult is mechanistically linked to the protection from other forms of regulated cell death, such as apoptosis and parthanatos. In ALS, HGF treatment prevents excitotoxic insult through the enhanced expression of glutamate transporter 1 in astrocytes and subsequent clearance of the excessive glutamate (Sun et al., 2002; Ishigaki et al., 2007), thus suggesting a role also in non-neuronal cells.

## HGF Exerts Antifibrotic Activity in Brain Injury

**FIGURE 5:**
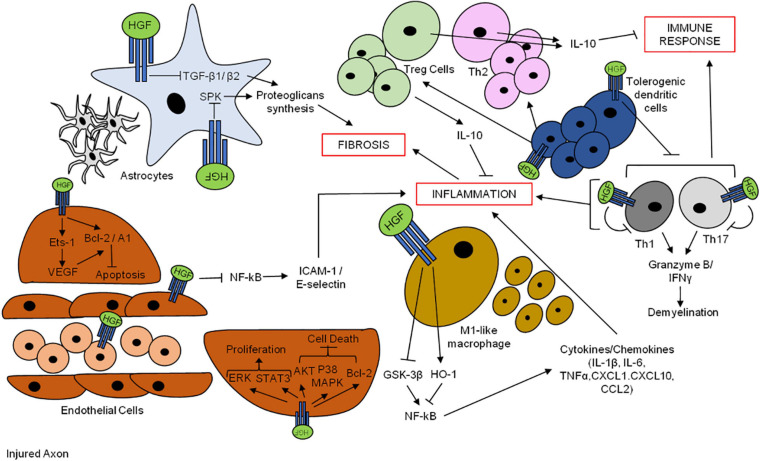
Hepatocyte growth factor promotes neuron survival after brain injuries. HGF-MET pair exerts anti-fibrotic and anti-inflammatory effects through the promotion of neuro-angiogenesis and modulation of the immune response.

Tissue damage after traumatic brain injury leads to the activation of astrocytes (Zhou et al., 2020; Figure 5). The activation and proliferation of glial cells, in turn, are useful in order to release signaling factors and to trigger a robust immune reaction consisting of brain-resident as well as peripherally recruited inflammatory cells. As the inflammatory response progresses, a glial scar is formed around the injury, consisting mostly of proteoglycans and reactive astrocytes. Activated astrocytes produce various proteoglycans, such as neurocan, phosphacan, and keratan-sulfate (McKeon et al., 1999; Zhang et al., 2006; Sofroniew, 2009), which are closely related to glial scar formation. In addition, transforming growth factor-β1 (TGF-β1), a potent fibrogenic protein, is known to upregulate astrocyte synthesis of proteoglycans causing severe gliosis (Yin et al., 2009; Schachtrup et al., 2010).

The process of scar formation probably exerts a role in protecting the integrity of the blood brain barrier (BBB) and in avoiding further cellular degeneration. However, formation of glial scar *via* the accumulation of reactive astrocytes is a major obstacle to axonal regeneration by neurons after injuries (Zhang and De Koninck, 2006; Fitch and Silver, 2008). Therefore, a critical issue for nerve regeneration and functional reconstruction is to inhibit the glial scar formation.

Exogenous HGF treatment shows an anti-fibrotic activity in animal models, such as the tMCAO model, exerted through the decrease of glial scar formation and scar thickness of the brain pia matter (Shang et al., 2011). HGF mostly exerts an anti-fibrotic effect on glial scar reduction by inhibiting the synthesis of the proteoglycans produced by the activated astrocytes through the complete block of TGF-β1 and TGF-β2 action. Indeed, the astrocyte activation and the glial scar formation were inhibited in a spinal cord injury (SCI) animal model transplanted with mesenchymal stem cells (MSCs) over-expressing HGF (Jeong et al., 2012). The transgenic overexpression of HGF in the nervous system attenuates spinal motoneuronal death and axonal degeneration, delays onset of the disease, and prolongs the lifespan of SOD1-G93A mice (Sun et al., 2002). A conditional *Met*-transgenic mouse was also generated, showing that the enhancement of MET levels in neurons selectively delays the disease onset in ALS mice models, prolonging lifespan, retarding motor neuron loss, and ameliorating motor performance (Genestine et al., 2011). HGF also reduces the number of astrocytes by retaining the levels of the glial-specific glutamate transporter 1, thus favoring a reduction in glutamatergic neurotoxicity (Sun et al., 2002). Moreover, HGF overexpression attenuates monocyte chemoattractant protein-1 (MCP-1) induction, predominantly in astrocytes, thus reducing accumulation of activated microglia (Kadoyama et al., 2007).

## HGF Induces Angiogenesis in the Nervous System

The ischemic stroke caused by reduced blood supply to specific areas of the brain leads not only to neuronal death but also to endothelial cell damage, which can result in the disruption of the BBB. Although HGF was originally identified as a potent mitogen for hepatocytes, it has also been recognized as a member of powerful pro-angiogenic growth factors (Bussolino, 1992).

Hepatocyte growth factor induces DNA synthesis and proliferation of vascular endothelial cells through the activation of ERK and STAT3 (Dong et al., 2001; Nakagami et al., 2001). HGF protects endothelial cells from hypoxic death by the AKT pathway and p38^*MAPK*^, as well as through the enhancement of Bcl-2 expression (Yamamoto et al., 2001; Wang et al., 2004; Figure 5).

Hepatocyte growth factor is also expressed in neuro-microvascular endothelial cells (Rosen et al., 1996), and an association between growth factors and increased angiogenesis in ischemic injuries is reported in literature. Angiogenesis could be a potent therapy for stroke patients by increasing the cerebral blood flow. Indeed, HGF amplifies angiogenesis after tMCAO in rats and reduces apoptosis *in vitro* (Shang et al., 2011). HGF protects endothelial cells against apoptosis in cerebral ischemia animal models and prevents the associated learning and memory dysfunctions (Date et al., 2004). HGF can also preserve the integrity of the BBB. Indeed, HGF protects BBB disruption after microsphere embolism-induced sustained cerebral ischemia by avoiding the inhibition of Bcl-2 (Date et al., 2006), thus decreasing apoptosis, and by reducing the expression of occludin and zonula occludens (ZO)-1, which are tight junction proteins, in cerebrovascular endothelial cells (Takeo et al., 2008). Concerning the molecular mechanisms implicated in HGF angiogenic role, there is a strong association with the E-twenty-six (Ets) pathway, which is implicated in the regulation of developmental and mitogen signals (Aoki et al., 2000). HGF can upregulate the transcription factor Ets-1, thus enhancing the expression of vascular endothelial growth factors (VEGFs) (Tomita et al., 2003). HGF and VEGF play a synergistic effect in promoting endothelial cell survival by enhancing the anti-apoptotic genes Bcl-2 and A1 (Xin et al., 2001). Moreover, HGF downregulates the expression of thrombospondin 1, which negatively regulates angiogenesis (Zhang et al., 2003; Scarpino et al., 2005).

## HGF Modulates Inflammation and Immune Response in the Brain

Local or systemic inflammation is a common response after insults such as tissue injuries and leads to secondary damages such as tissue destruction. Neutrophils, eosinophils, and macrophages are the principal actors in promoting the inflammatory response.

Hepatocyte growth factor regulates the inflammatory and immune response in many pathological conditions by modulating cell migration, antigen presentation, T-cell effector function, and cytokine production, thus exerting a protective function (Figure 5).

Hepatocyte growth factor decreases nuclear factor kappa-light-chain-enhancer of activated B cells (NF-κB) activation to downregulate adhesion molecules such as ICAM-1/E-selectin on the endothelial cell surface (Makondo et al., 2004; Mizuno and Nakamura, 2005), thus inhibiting neutrophil infiltration and suppressing ischemia-related injury in various organs (Li et al., 2007; Makiuchi et al., 2007; Ohnishi et al., 2008). Moreover, HGF is able to directly target activated eosinophils through the downregulation of inflammatory mediators during allergic response (Ito et al., 2005).

In the context of brain injury, macrophages exert a pivotal role in the induction of the inflammatory response (Popovich et al., 1999). The balance between two subsets of macrophages is known: the M1-like macrophages, which are associated with antimicrobial and pro-inflammatory activity, and the M2-like macrophages, which are mostly involved in tissue repair and are associated with an immune-modulatory activity.

Hepatocyte growth factor ameliorates tissue damage and functional recovery after the acute phase in a mouse model of SCI by exerting anti-inflammatory effects through the diminution of LPS-activated macrophages and neutrophil infiltration (Yamane et al., 2018). Mechanistically, HGF inhibits inflammatory M1-like macrophage production of pro-inflammatory cytokines and chemokines (such as IL-1β, IL-6, TNFα, CXCL1, CXCL10, and CCL2) by disrupting nuclear factor NF-κB signaling (Giannopoulou et al., 2008) through the enhancement of the heme oxygenase-1 (HO-1) transcriptional pathway and the inactivation of the GSK-3β pathway (Kamimoto et al., 2009a,b; Coudriet et al., 2010).

It has also been shown that HGF stimulates macrophage differentiation in dendritic immune-suppressive cells, thus facilitating the induction of T lymphocyte regulators, associated with a pivotal role in anti-inflammation. There is large evidence that HGF can mediate neuroinflammation in the context of neurodegenerative autoimmune diseases such as multiple sclerosis (MS), leading to an indirect effect on the immune system. In a study by Benkoucha et al., HGF exerted an anti-inflammatory effect through the generation of tolerogenic dendritic cells with the consequent suppression of auto-reactive T helper cells 1 (Th1) and Th17 cells and leading to the reduction of CD4 + T-cell-mediated nervous system injury in the experimental autoimmune encephalomyelitis (EAE) animal model of MS, in which HGF was overexpressed (HGF transgenic mice) (Benkhoucha et al., 2010). The authors also showed, later, that the administration of HGF or adoptive transfer of HGF-treated dendritic cells in recipient EAE mice reduces disease progression and induces dendritic tolerance. They observed an increase in the frequency of both peripheral and central nervous system anti-inflammatory IL-10-secreting regulatory T cells (Treg cells) and, in parallel, a decrease of pro-inflammatory Th1 and Th17 responses (Benkhoucha et al., 2014). Accordingly, a study by Bai et al. demonstrated a functional benefit in EAE mice after MSC transplantation or exogenous recombinant HGF supply, associated with a reduction in the severity of the disease. Moreover, pre-treatment with anti-MET antibodies blocked the beneficial effect exerted by HGF, while the one exerted by MSCs was blocked by anti-HGF antibody, showing that the positive effect was mostly dependent on the production of HGF. In addition, both MSCs and HGF stimulated the development of neurons and the proliferation and migration of myelinating oligodendrocytes (OLs), reduced pro-inflammatory cytokines, and enhanced anti-inflammatory cytokines. Importantly, HGF also promoted re-myelination *in vitro* (Bai et al., 2012).

On the basis of these studies, the capacity of HGF to modulate neuro-inflammation is thus associated with an alteration in the balance of pro-inflammatory (Th1 and Th17) and anti-inflammatory (Th2 and Tregs) CD4 + T cells by influencing dendritic cell function. Recently, Benkoucha et al. found that MET is also expressed in a subgroup of the encephalogenic CD8 + cell population, which is significantly increased at the peak phase of clinical disease in EAE immunized mice and decreased at the recovery stage. CD8 + cells recognize the encephalitogenic epitope myelin OL protein_35__–__50_ (MOG_35__–__50_) and induce demyelination through the secretion of granzyme B, lymphotoxin, and IFNγ (Buckle et al., 2003; Ifergan et al., 2011). HGF was shown to decrease the effector functions of these MET + CD8 + cells in EAE mice by decreasing granzyme B and IFNγ levels (Benkhoucha et al., 2020). This study suggests that, on one side, expression of MET in CD8 + T cells may have a role in the causality of central nervous system pathology but, on the other side, HGF, through unknown mechanisms, may control this highly cytotoxic MET + CD8 + T-cell population.

## HGF and Stem Cell Therapies for Neuroregeneration

**FIGURE 6:**
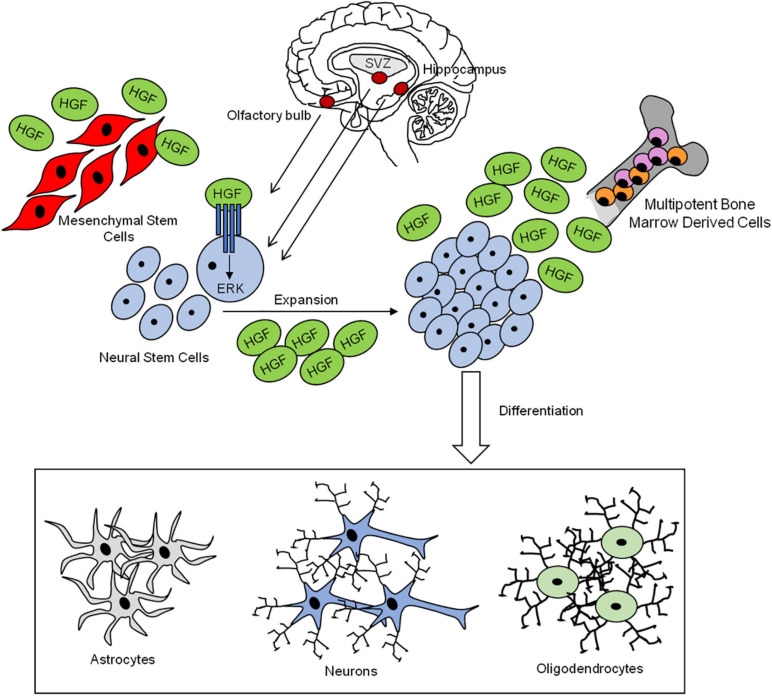
Neural stem cells contribute to nervous system cell regeneration. HGF-MET pair drives the mobilization, expansion, and differentiation of neural stem cells into the three main nervous system cells: astrocytes, neurons, and oligodendrocytes. HGF is secreted by bone marrow-derived stem cells and mesenchymal stem cells.

Neural stem cells (NSCs) are the stem cells of the nervous system, to which they give birth during development. Many progresses have been obtained in the last decades in the comprehension of their biology. A small number of NSCs remain in adults even if most of them are quiescent (recently reviewed in Zhao and Moore, 2018). Despite that, for a long time, it was believed that no neurons are made in adult brains, the evidence that neurogenesis is also possible in human adult in the SVZ, in the sub-granular zone (SGZ) on the hippocampal dentate gyrus (DG), and in the olfactory bulb (Braun and Jessberger, 2014; reviewed in Brann and Firestein, 2014; Zhao and Moore, 2018) strikingly reversed that dogma, opening the possibility to employ stem cells as new tools for therapeutic purposes in neural regeneration/repair in diseases and after injuries (Figure 6).

Neural stem cells own self-renewal ability, and they can differentiate into three types of cells, like neurons, OLs, and astrocytes. The regulation of NSC proliferation, self-renewal, and differentiation is finely regulated by changes in the surrounding microenvironment, or niche, in which NSCs reside (reviewed in Andreotti et al., 2019).

Hepatocyte growth factor contributes to the maintenance of self-renewal and proliferation of NSCs in SVZ, which express MET and produce HGF. HGF promotes self-renewal and expansion of NSCs forming neurospheres. Mechanistically, the mitogenic effect promoted by HGF in SVZ cells is dependent on the activation of the ERK pathway (Nicoleau et al., 2009).

Previous reports in the context of SCI recovery reported a positive effect of HGF in the promotion of grafted NSC differentiation and synapse formation between new neurons and the descending cortico-spinal fibers (Kokuzawa et al., 2003; Kato et al., 2004; Takano et al., 2017).

Neural stem cell differentiation into astrocytes and, at a lower extent, into new neurons is regulated by some factors such as bone marrow proteins (BMPs), whose expression is upregulated in the injury lesion after SCI (Wang et al., 2011; Sandner et al., 2013). BMPs regulate NSC differentiation into astrocytes by acting through the phosphorylation of Smad proteins 1, 5, and 8, which combine with Smad4 in order to form a complex that translocates to the nucleus and regulates gene transcription (Itoh et al., 2000; Sandner et al., 2013). The BMP/Smad pathway effect is counteracted by the transplantation of MSCs derived from bone marrow (BMSCs), which promote NSC differentiation into OLs, the myelinating glia of the CNS (Sandner et al., 2013; Song et al., 2018). A recent study on SCI rats showed that BMSCs probably exert their biological effect through HGF-MET axis activation. HGF inhibits the BMP/Smad pathway and promotes the differentiation of NSC neurospheres into neurons and axon outgrowth *in vitro* similarly to BMSCs and in a dose-dependent fashion. Moreover, the BMSC-associated effect is inhibited by anti-MET antibodies. Therefore, the authors hypothesized that the beneficial effect exerted by BMSCs in the nervous system might depend on HGF secretion and on HGF-MET axis activation (Song et al., 2020). HGF also promotes differentiation of BMSCs into neuron-like cells in the context of SCI, in combination with glial cell-derived neurotrophic factor (GDNF) (Zheng et al., 2013; Ma et al., 2016).

Mesenchymal stem cell transplantation exerts a beneficial role also in the EAE mice, the animal MS model, promoting a functional recovery (Bai et al., 2012). In this study, the ability of MSCs to improve EAE model by re-myelination and to reduce inflammation was associated with HGF secretion and MET activation. Indeed, the anti-MET and anti-HGF-neutralizing antibodies block the beneficial effects of both MSCs and HGF in EAE. In addition, the authors show that HGF accelerates re-myelination of lysolecithin-induced demyelinated spinal cord (Bai et al., 2012). In another study, employing the MPP + PD animal model, the supernatant from human umbilical cord (hUC) MSCs infected by an adenovirus carrying the HGF gene promotes a better regeneration of damaged PD dopaminergic neurons as compared to hUC-MSCs alone. Mechanistically, this beneficial effect seems to be mediated by the regulation of intracellular Ca^2+^ levels by the modulation of calbindin expression, which is associated with calcium homeostasis and plays a role in the protection of dopaminergic neurons in the pathogenesis of PD (Liu et al., 2014). A recent study shows that hUC-MSCs are also useful for the recovery of cognitive ability in a mouse model of AD and that the main actor in the beneficial effect is HGF. This beneficial effect is, at least in part, due to the activation of the MET-AKT/GSK-3β signaling pathway (Jia et al., 2020).

The influence mediated by HGF secretion and HGF-induced MET activation in all these studies provides an evidence that, beyond stem-cell ability to differentiate into neurons, the secretion of paracrine growth factors such as HGF provides an important regenerative capacity and a promising therapeutic tool. Thus, HGF, as a paracrine factor, may contribute to successful stem-cell-based therapies, which have been investigated in many clinical trials in the context of pathologies such as SCI, PD, ALS, AD, and MS with promising results (Jafarzadeh Bejargafshe et al., 2019; Staff et al., 2019; Platt et al., 2020; Silvestro et al., 2020).

Hepatocyte growth factor also acts on non-neural cells in the context of peripheral nerve regeneration, such as after a peripheral nerve injury. When peripheral nerves get wounded, the myelin structures of Schwann cells (SCs) dedifferentiate and begin the regeneration process (Gaudet et al., 2011).

Levels of HGF are increased at the injured and distal sites in a mouse model of sciatic nerve injury, and MET and pMET are upregulated mostly in distal SCs. HGF administration promotes the migration and proliferation of cultured SCs and induces the expression of various neurotrophic factors (Ko et al., 2018). Indeed, HGF controls SC migration in cooperation with neuregulin-1 through the regulation of Gab2. HGF phosphorylates MET, ERK, and AKT (Shin et al., 2017; Ko et al., 2018) in a sciatic nerve explants, and the MET inhibitor crizotinib blocks Gab2 phosphorylation in SCs (Shin et al., 2017). Furthermore, endogenous HGF supply *in vivo* in the periphery injury mouse model enhances re-myelination. Accordingly, HGF and MET play an important role in SC-mediated nerve repair (Ko et al., 2018).

## Therapeutical Potential of HGF and HGF Mimetics in Neurological Disorders

Hepatocyte growth factor promotes beneficial effects in several animal models of neurological diseases, as indicated in the previous paragraph by studies with HGF-secreting stem cell transplantation. In this section, we report some of the most important results reached through HGF or HGF mimetic administration in a panel of animal models of neurological diseases (Tables 1, 2) and some outcomes from human clinical trials (Table 3). HGF protein benefits are largely dependent on the way of administration, mostly due to HGF short half-life and poor accessibility to the BBB. In this view, alternative tools were also developed in order to overcome these problems and ameliorate the delivery of HGF or MET agonists to the desired target. Topical injections of recombinant HGF have been successfully employed in many animal models of neurological diseases. Continuous intrathecal recombinant human HGF injections exert functional recovery in non-human primate model of SCI (Aoki et al., 2019) and protect from neuronal death in ALS mice models (Ishigaki et al., 2007). A clinical trial (UMIN000007062) on ALS patients recently evaluated the safety and efficacy of a five-residue-deleted human HGF (KP-100) administered intrathecally. Positive outcomes emerged, but further studies are needed in order to validate its clinical efficacy (Warita et al., 2019). The intrathecal administration of HGF also exerts positive functional recovery and protection of myelinated areas in animal models of SCI (Kitamura et al., 2011). Its efficacy was evaluated in a clinical trial (NCT02193334) in patients with acute SCI, with encouraging motor functional recovery (Nagoshi et al., 2020). Moreover, intrastriatal injections of HGF were successful in protecting neural progenitor cells from apoptosis in animal models of cerebral ischemia (Doeppner et al., 2011). Another way to deliver HGF is the use of hydrogel carriers, which are useful in supporting neurogenesis (Nakaguchi et al., 2012) and mitigate apoptosis and autophagy (Shang et al., 2010) after stroke in mice models. This therapeutic strategy was also successful in promoting neuronal regeneration and reducing inflammation in a recent study on SCI mice model (Yamane et al., 2018).

**TABLE 1:** Administration of HGF and HGF mimetics in animal models of neurological diseases.

Disease application	Methods	Year	Animal model	Outcomes	References
ALS	Continuous intrathecal delivery of rhHGF in transgenic rats (SOD1-G93A)	2007	Rat	Inhibition of caspase-3 and -9 induced apoptosis in motor neurons and XIAP levels enhancement; enhancement of EAAT2 protein in astrocytes	Ishigaki et al., 2007
	Continuous administration of rhHGF intrathecally after a cervical spinal cord injury	2019	Marmoset	Functional recovery promotion	Aoki et al., 2019
	Intraperitoneal administration of K1K1 in transgenic rats (SOD1-G93A)	2020	Mouse	Rescue of motor neurons from death in spinal-neuron astrocyte co-cultures and in the lumbar spinal cord of transgenic mice; transient amelioration of the muscle force impairment	Vallarola et al., 2020
AD	Intraperitoneal injection of the HGF mimetic dihexa in the scopolamine amnesia and aged-related models	2013	Rat	Antidementia potential; dendritic spinogenesis augmentation in the hippocampus	McCoy et al., 2013
SCI	Intrathecal rhHGF infusion during the acute phase of cervical SCI	2011	Marmoset	Functional recovery; cortical fibers and myelinated area protection	Kitamura et al., 2011
	Engineered HGF with a collagen binding domain (CBD-HGF) and photo-cross-linkable gelatin-gelatin-furfurylamine	2018	Mouse	Axonal regeneration promotion; motor recovery; neutrophil and macrophage reduction	Yamane et al., 2018
Cerebral ischemia	Sponge soaked with rhHGF in tMCAO models	2010	Rat	Modulation of autophagy and apoptosis	Shang et al., 2010
	Acute intra-striatal injection of HGF in a stroke model	2011	Mouse	Proliferation of neural progenitor cells and protection from caspase-induced death; reduction of post-ischemic functional deficits; diminution of the BBB permeability by MMP inactivation	Doeppner et al., 2011
	Gelatin hydrogel microspheres containing HGF in MCAO stroke model	2012	Mouse	Increased number of new neurons migrating from the SVZ toward the injured striatum in a stroke model in adult mice	Nakaguchi et al., 2012
	BB3 administration in tMCAO or pMCAO models for 14 days	2015	Rats	Long-term neurologic function improvement; little effect on cerebral infarct size reduction	Chaparro et al., 2015

*AD, Alzheimer’s disease; ALS, amyotrophic lateral sclerosis; BBB, blood brain barrier; EAAT2, excitatory amino acids transporter 2; MCAO, middle cerebral artery occlusion; MMP, matrix metallo-proteinases; PD, Parkinson’s disease; pMCAO, permanent middle cerebral artery occlusion; rhHGF, recombinant human hepatocyte growth factor; SCI, spinal cord injury; SVZ, sub-ventricular zone; tMCAO, transient middle cerebral artery occlusion; XIAP, X-linked inhibitor of apoptosis protein.*

**TABLE 2:** Hepatocyte growth factor gene transfer in animal models of neurological diseases.

Disease application	Methods	Year	Animal model	Outcomes	References
ALS	Generation of HGF transgenic mice (SOD1-G93A)	2002	Mouse	Inhibition of caspase-1 induced apoptosis and reduction of iNOS in motor neurons; protection from excitotoxic death induced by glutamate; EAAT2 protein enhancement in astrocytes; microgliosis and astrocytosis reduction	Sun et al., 2002
	Intrathecal injection of r-AAV HGF in SOD1-G93A mice	2019	Mouse	Motor neuron protection and delay in degeneration; ERK activation implication	Lee et al., 2019
PD	*In vivo* naked HGF plasmid DNA transfer into the striatum in 6-OHDA models	2006	Rat	Protection of dopaminergic neurons from neuron death; inhibition of abnormal amphetamine-induced rotation	Koike et al., 2006
	*In vivo* naked HGF plasmid DNA transfer in the putamen using a stereotaxic technique in the MPTP model	2009	Monkey	Prevention of abnormal amphetamine-induced rotation in hemi-PD primates; gradual reduction of limb tremor and rigidity; prevention (reparation) of dopaminergic neuron MPTP-induced death	Koike et al., 2009
	Overexpression of hHGF plasmid DNA using ultrasound-mediated gene transfer into the brain in an Aβ-infused cognitive dysfunction mouse model.	2008	Mouse	Amelioration Aβ-induced memory impairment; high recovery of vessel density in the hippocampus; upregulation of BDNF; significant decrease in oxidative stress; synaptic enhancement.	Takeuchi et al., 2008
SCI	Exogenous HGF administration using a replication-incompetent HSV-1 vector	2007	Mouse	Promotion of neuron and oligodendrocyte survival by the reduction of cleaved caspase-3 activation; angiogenesis promotion; demyelination reduction	Kitamura et al., 2007
Peripheral nerve injury	Intramuscular injection of the HGF plasmid pCK-HGF-X7 around the sciatic nerve	2018	Mouse	Increase in myelin thickness and axon diameter	Ko et al., 2018
	Intramuscular injection of the plasmid pC4W-hHGF bearing human HGF	2018	Mouse	Nerve structure restoration; functional recovery; inflammation decrease	Boldyreva et al., 2018
Neurophatic pain	HGF gene transfer transfected by the non-viral HVJ liposome administered by repeated intramuscular injection in the CCI model	2008	Rat	Pain relief; reduction of P2 × 3, P2 × 4, and P2Y1 receptor mRNA levels, and of IL-6 and ATF3 mRNAs induced by CCI	Shimamura et al., 2006
	HGF plasmid DNA (VM202 or pCK-HGF-X7) administered by intramuscular injection in chronic constriction injury (CCI) model	2018	Mouse	Neuropathic pain suppression for several weeks; downregulation of the expression of pain-related markers; reduction in the number of activated microglia and astrocytes	Nho et al., 2018
Cerebral ischemia	HGF gene transfer by the non-viral HVJ liposome	2001	Mongolian gerbils	Prevention of neuronal cell death	Hayashi et al., 2001
	HGF gene transfer by the HVJ-envelope	2006	Rat	Increase of synaptogenesis; neurite extension promotion; prevention of gliosis	Shimamura et al., 2006

*ALS, amyotrophic lateral sclerosis; ATF3, activating transcription factor 3; BDNF, brain-derived neurotrophic factor; CCI, chronic constriction injury; EAAT2, excitatory amino acids transporter 2; HGF, hepatocyte growth factor; HJV, hemagglutinating virus of Japan; HSV-1, herpes simplex virus-1; IL-6, interleukin 6; iNOS, inducible nitric oxide synthase; MPTP, 1-methyl-4-phenyl-1,2,3,6-tetrahydropyridine; PD, Parkinson’s disease; P2 × 3, P2X purinoreceptor 3; P2 × 4, P2X purinoreceptor 4; P2Y1, P2Y purinoreceptor 1; r-AAV HGF, recombinant adeno-associated virus encoding human hepatocyte growth factor; SCI, spinal cord injury; 6-OHDA, 6-hydroxydopamine.*

**TABLE 3:** Human clinical trials of HGF-based therapy in neurological diseases.

Disease application	Method	Phase	Clinical Trial	Year	Outcomes	References
SCI	Intrathecal injection of KP-100IT	I/II	NCT02193334	2020	No harmful adverse effects during observational period; motor functional recovery (motor score improvement)	Nagoshi et al., 2020
Diabetic neuropathy	Intramuscular injection of VM-202 plasmid	II	NCT01475786	2015	No serious adverse effects; symptomatic relief with improvement in quality of life for 3 months	Kessler et al., 2015
AD	Subcutaneous injection of NDX-1017 (or ATH-1017)	I	NCT03298672	2017	No serious adverse effects; dose-dependent and consistent amelioration in brain network activity across all treated cohorts	-
ALS	Intramuscular injection of VM-202 plasmid on various muscular groups	I/II	NCT02039401	2017	No serious adverse effects; larger study needed to assess the efficacy	Sufit et al., 2017
	Intrathecal injection of 5-residue-deleted HGF through an implantable catheter connected to a subcutaneous por	I	UMIN000007062	2019	No serious adverse effects	Warita et al., 2019

*AD, Alzheimer’s disease; ALS, amyotrophic lateral sclerosis; HGF, hepatocyte growth factor; SCI, spinal cord injury.*

A preferred strategy is the delivery of exogenous HGF by gene transfer, with the advantage of less side effects. HGF gene therapy employing naked plasmids succeeds in preventing neuronal death in animal models of ALS (Sun et al., 2002) and PD (Koike et al., 2006, 2009) and induces analgesic effects in a neuropathic pain model (Nho et al., 2018). The latter results have encouraged the clinical evaluation of HGF (VM-202) gene transfer in human trials for neuropathic diabetic pain (NCT01475786) with the encouraging outcome of a 3-month pain relief period (Kessler et al., 2015). The same HGF plasmid (VM-202) was also evaluated in ALS patients in order to define its tolerability and safety profile (NCT02039401). Even if no adverse effects were detected, a larger study will be necessary in order to validate its clinical efficacy (Sufit et al., 2017). HGF gene therapy also promotes re-myelination and the reduction of inflammation in peripheral nerve injury models (Boldyreva et al., 2018; Ko et al., 2018). Moreover, viral vector gene transfer has been tested pre-clinically and is more promising because it provides a more efficient transcription transfer than non-viral methods. Thus, gene therapy with viral vectors protects from neuronal death in animal models of ALS (Lee et al., 2019) and cerebral ischemia (Hayashi et al., 2001), promotes angiogenesis, and reduces apoptosis in SCI (Kitamura et al., 2007). Moreover, HGF gene transfer with viral vectors reduces pain and inflammation in a neuropathic model (Tsuchihara et al., 2008), promotes neurite extension, and increases synaptogenesis in a stroke model (Shimamura et al., 2006). A further strategy is the overexpression of hHGF plasmid DNA using ultrasound-mediated gene transfer. By this delivery method, HGF improves angiogenesis, decreases oxidative stress, enhances synaptogenesis, and increases BDNF levels in the brain of AD mouse model (Takeuchi et al., 2008).

Finally, HGF mimetic compounds have been developed and own less difficulties than gene transfer and protein therapies. Dihexa (N-hexanoic-Tyr-Ile-(6) aminohexanoic amide), belonging to AngIV-related peptides, is an allosteric activator of HGF and enhances MET signaling in the brain (Benoist et al., 2014). Dihexa reduces dementia in AD models (McCoy et al., 2013). This compound crosses the BBB and does not need to be manufactured by recombinant methods at high cost compared to HGF, which represents an obvious advantage. Another BBB-crossing molecule with HGF-like activity, BB3, was employed in pre-clinical models of stroke, showing good functional improvements (Chaparro et al., 2015). A small molecule enhancing MET activity (the MET agonist NDX-1017 or ATH-1017) was recently tested in AD patients (NCT03298672), resulting in positive outcomes in terms of brain network activity, even if larger studies need to be performed to show more robust and statistically significant results. Molecules capable of inducing MET receptor dimerization and activation were sought. These MET agonists include anti-MET monoclonal antibodies based on the bivalent characteristic of antibodies (Prat et al., 1998; Pietronave et al., 2010; Gallo et al., 2014a; Yuan et al., 2019), the engineered protein eNK1, created by the disulfide-linked NK1 (N-terminal and the K1 first kringle) domains of HGF (Jones et al., 2011; Liu et al., 2014), a novel dimeric form of the K1 domain, designated K1K1 (Vallarola et al., 2020), the semi-synthetic engineered K1 domain, designed to be multimer by biotin-avidin interaction (Simonneau et al., 2015), a MET-agonistic DNA aptamer (Ueki et al., 2016, 2020), and MET-agonistic synthetic macrocyclic peptides (Sato et al., 2020). All these MET agonists created by different strategies are expected to augment the therapeutic armamentarium for brain repair and neurological and neurodegenerative diseases. In particular, the intraperitoneal administration of K1K1 resulted in a strong neuroprotective activity on motor neurons in ALS animal models (Vallarola et al., 2020).

## Conclusion

Cell survival is a relevant biological response to HGF-MET signaling; it endures development and regeneration of brain and nerve cells and can be exploited for therapy. HGF and MET expression has been detected in neural tissues during development, and it has been associated with neuron wiring and synaptogenesis. A genetic implication of HGF and MET in the development of brain emerges from the literature, and various alterations in the HGF-MET pathway correlate with features of some NDDs such as ASD, schizophrenia, and non-syndromic hearing loss. Nevertheless, the molecular mechanism linking *MET* and *HGF* mutations to these disorders is still poorly understood and is the topic of intense investigation.

Hepatocyte growth factor treatment exerts beneficial effects in the context of brain ischemia and injuries and in a number of neurodegenerative diseases in animal models. HGF protects neurons and promotes their survival via anti-apoptotic, anti-fibrotic, anti-inflammatory, pro-angiogenic, and immune-modulatory actions.

However, despite its promising therapeutic effects, the use of HGF protein is limited by its poor bioavailability. Some strategies, such as agonist HGF monoclonal antibodies, HGF gene transfer therapy, and HGF mimetics, may overcome the pharmacokinetic limitations. Human clinical trials are ongoing, showing, so far, good safety and tolerability.

## Author Contributions

CD performed the bibliography study and wrote the manuscript review. SG and AV collaborated to text and figures. TC and PC conceptualized the review and revised it together with AB, FM, and DV. All authors agree to be accountable for the content of the work.

## Conflict of Interest

PC is a founder of the Benefit Company ‘METIS’. The remaining authors declare that the research was conducted in the absence of any commercial or financial relationships that could be construed as a potential conflict of interest. The reviewer GM declared a shared affiliation, with no collaboration, with the authors to the handling editor at the time of the review.

## Abbreviations

AD: Alzheimer’s disease
AIF: apoptosis-inducing factor
ALS: amyotrophic lateral sclerosis
AMPA: α-amino -3-hydroxy-5-methyl-4-isoxazolepropionic acid
APE/Ref-1: apurinic/apyrimidinic endonuclease/redox factor-1
ASD: autism spectrum disorder
cKO: conditional knock-out
BBB: blood brain barrier
BMSC: bone marrow derived stem cell
CREB: cAMP response element-binding protein
EAAT-2: excitatory amino acids transporter 2
EAE: experimental autoimmune encephalomyelitis
ERK: extracellular signal regulated kinase
Ets: E-twenty -six
GSK-3 β: glycogen synthase kinase 3 beta
HGF: hepatocyte growth factor
HO-1: heme oxygenase-1
hUC-MSC: human umbilical cord mesenchymal stem cell
LTD: long-term depression
JNK: c-JunN-terminal protein kinase
LTP: long-term potentiation
MAPK: mitogen-activated protein kinase
MAP2: microtubule-associated 2 protein
MS: multiple sclerosis
MSC: mesenchymal stem cell
mTOR: mammalian target of rapamycin
NDD: neurodevelopmental disorders
NF- κ B: nuclear factor kappa-light-chain-enhancer of activated B cells
NGF: nerve growth factor
NMDA: N-methyl -D-aspartate
NSC: neural stem cell
NSHI: non-syndromic hearing loss
OL: oligodendrocyte
PD: Parkinson’s disease
PI-3K: phosphoinositide-3-kinase
PLC γ: phospholipase c gamma
pMET: phospho-MET
Rac1: C3 botulinum toxin substrate 1
Ras: rat sarcoma
SC: Schwann cell
SCG: superior cervical ganglion
SCI: spinal cord injury
SNP: single-nucleotide polymorphism
SPH: serine proteinase homology
SPK: sphingosine kinase
STAT3: signal transducer and activator of transcription 3
SVZ: sub-ventricular zone
TGF: transforming growth factor
Th cell: T helper cell
tMCAO: transient middle cerebral artery occlusion
Treg cell: regulatory T cell
uPAR: urokinase plasminogen activator receptor
VEGF: vascular endothelial growth factor.
